# Behavior of an Advanced Bolted Shear Connector in Prefabricated Steel-Concrete Composite Beams

**DOI:** 10.3390/ma12182958

**Published:** 2019-09-12

**Authors:** Jun Chen, Wei Wang, Fa-Xing Ding, Ping Xiang, Yu-Jie Yu, Xue-Mei Liu, Fu Xu, Cai-Qian Yang, Shi-Guo Long

**Affiliations:** 1 College of Civil Engineering and Mechanics, Xiangtan University, Xiangtan 411105, China; chenjun0325@126.com (J.C.); wwei5633@126.com (W.W.); xufu@xtu.edu.cn (F.X.); longsg@xtu.edu.cn (S.-G.L.); 2 School of Transportation, Wuhan University of Technology, Wuhan 430063, China; 3 School of Civil Engineering, Central South University, Changsha 410075, China; yujiecsu@csu.edu.cn; 4 Engineering Technology Research Center for Prefabricated Construction Industrialization of Hunan Province, Changsha 410075, China; 5 Department of Infrastructure Engineering, The University of Melbourne, Parkville, VIC 3010, Australia; xuemei.liu@unimelb.edu.au; 6 College of Civil Engineering, Southeast University, Nanjing 210018, China

**Keywords:** prefabricated steel-concrete composite beam, high-strength bolt connector, push-off test, finite element model, shear bearing capacity

## Abstract

The high-strength bolt shear connector in prefabricated concrete slab has advantages in applications as it reduces time during the construction of steel-concrete composite building structures and bridges. In this research, an innovative and advanced bolt shear connector in steel-concrete composite structures is proposed. To investigate the fundamental mechanical behavior and the damage form, 22 static push-off tests were conducted with consideration of different bolt dimensions, the reserved hole constraint condition, and the dimension of slab holes. A finite element (FE) model was established and verified by using test results, and then the model was utilized to investigate the influence of concrete strength, bolt dimension, yield strength, bolt pretension, as well as length-to-diameter ratio of high strength bolts on the performances of shear connectors. On the basis of FE simulation and test results, new design formulas for the calculation of shear resistance behavior were proposed, and comparisons were made with current standards, including AISC, EN 1994-1-1, GB 50017-2017, and relevant references, to check the calculation efficiency. It is confirmed that the proposed equation is in better agreement with the experimental results.

## 1. Introduction

**Figure 1:**
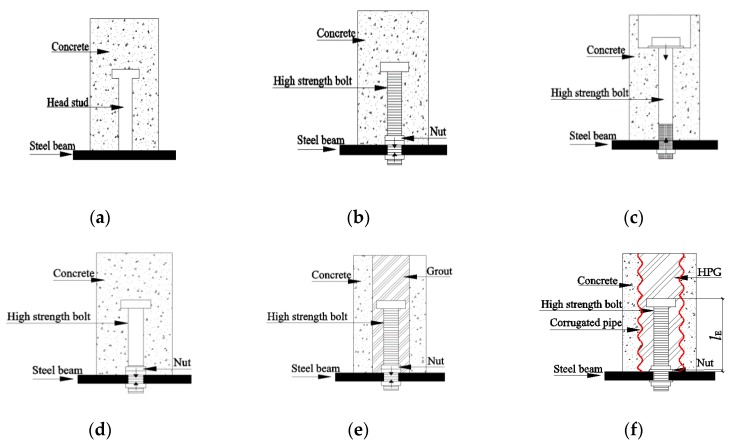
Welded headed stud and different types of bolt shear connectors: (**a**) headed stud; (**b**) Type 1; (**c**) Type 2; (**d**) Type 3; (**e**) Type 4; (**f**) Type 5.

A mechanical shear connector is an essential component of composite steel-concrete beams to ensure stability [1]. A shear connector is applied to transfer the shear forces across steel beams and concrete slabs. At present, many types of steel-concrete connectors have been developed, and the weld headed connector is most commonly used owing to its good performance in welding (Figure 1a). Comprehensive studies on shear performance and the mechanisms of this shear connector have been conducted [2,3,4,5]. Results based on those studies showed that shear capacity of headed stud connectors is governed by the concrete strength, and the dimension and yield strength of shear studs.

In recent decades, prefabricated structures have been widely built as they make construction environmentally-friendly and efficient [6,7,8]. Reliability of connections between prefabricated elements is the governing factor in maintaining the structural integrity and continuity. The mechanical couplers can be designed and used in prefabricated column-to-cap and column-to-foundation connections, such as grouted sleeve connectors, aluminum tubes, and cylindrical pipes, to strengthen the anchorage bond and shorten embedment length [9,10,11]. Due to the rapid development of prefabricated structures, special attention has been paid to the high-strength bolt shear connectors. High-strength bolt shear connectors (Figure 1b–e), are more suited for the use in construction of composite beams than traditional ones as they are easily and efficiently dismantled and assembled. Dallam [12] investigated the static push-out tests of high-strength bolted shear connectors, and the results revealed that the ultimate shear capacity of bolted shear connections was greater than that of the headed shear studs, and shear capacity of the former type increased with the increase of bolt diameter. The push-out tests on two types of high-strength bolts (Figure 1c,e) were conducted by Dedic and Klaiber [13], and it was found that the shear capacity of high-strength bolt connectors would be underestimated when analyzed by the shear formulas for stud headed connector suggested by AASHTO code. Kwon et al. [14] investigated the static and fatigue behavior of three types of post-installed shear connectors, namely Double Nut Bolt (Figure 1b), High-Tension Friction-Grip Bolt (Figure 1c), and Adhesive Anchor. Then, calculation formulas were proposed under different loading conditions, but the study aimed to strengthen the existing structures rather than sustainable construction. High-tension friction grip bolt shear connectors (Figure 1c) with precast Geopolymer concrete (GPC) slab were investigated by Ataei et al. [15] and Liu et al. [16,17] through comprehensive experiments and finite element (FE) analysis. Then on the basis of on simulation and push-out test data, a design formula was proposed, without regarding the influence of concrete strength. Studies on the static shear resistance of high-strength bolt composite beams, were conducted by Moynihan and Allwood [18] with three types of beams of 2, 5, and 10 m long specimens to be tested. It was observed that the formula in Eurocode 4 [19], which was designed to predict the shear capacity of headed studs, would produce conservative predictions for the high-strength bolt shear connectors. The potential and suitability of the demountable shear connectors developed from headed stud were investigated by Dai et al. [7] through push-out tests and the results demonstrated that proposed bolted connectors behaved similarly in mechanism as headed studs. Pavlovic et al. [20] explored the difference between mechanical performance of high strength bolt (Figure 1d) and that of headed stud shear connectors through push-out tests and FE analysis. In studies conducted by Suwaed and Karavasilis [21] a novel demountable bolted shear connector was presented to be suitable for application in composite bridges as it can be decommissioned when any components break down. Additionally, the research results also indicated that the bolted shear was advantageous for use owing to its higher shear capacity, stiffness, and ductility than headed studs. In the Type 1 bolted shear connector, the preloading is only induced on the bolt shank between upper and bottom nuts. In the Type 2 bolted shear connector, the compressive stress of concrete slab near the bolt shank may be high, because of the preloading of the high strength bolt. In Type 3, a demountable shear connector is improved by the headed stud. The Type 4 bolted shear connector is the same as the Type 2, except the concrete slab is prefabricated.

In the prefabricated steel-concrete composite beams, the high-strength bolts, concrete slabs, steel beams and in-filled materials are the major components of the high-strength bolt connectors, and mechanical behavior of the shear connectors is affected by these factors. Previous studies on bolted shear connectors mainly focused on traditional factors such as the bolt diameter, concrete strength, and bolt types. While the effect of concrete quality around those high strength bolts were rarely reported, in practical conditions, the concrete compaction around the high strength bolts may not be as good as that around the headed studs due to the thread construction, especially for the Type 1 and Type 4 connectors in Figure 1.

In this paper, an advanced high strength bolted shear connector design was proposed (see Figure 1f). This connector featured a corrugated pipe instead of ordinary hole. In this way, the corrugated nature can strengthen the bonding mechanism between post-grouted material and precast slab.

## 2. Advanced Bolt Shear Connectors

Figure 1f showes the schematic diagram of the proposed advanced bolt shear connectors. This bolted connector consists of a prefabricated slab with an embedded corrugated pipe, high-strength bolt, and steel beam, which can be disassembled and re-assembled, and all these components can be customized in a factory. Compared with other types of bolt connectors, this bolted shear connector have some advantages: (1) construction convenience: in the case of grouting, the traditional connectors (Type 4) used pre-embedded circular steel tubes to create the spared hole, and the tube needed to be taken out before grouting. While for the novel bolt shear connector, the spared corrugated pipe holes did not need to be taken out and the grouting material can be filled directly; (2) High bonding mechanism: due to the application of corrugated pipes and high strength pouring material (HPG), the compactness of the grouting material can be improved. Additionally, the bonding mechanism to precast the slab portion was also stronger due to the corrugated construction and the large contact area between the surface of corrugated pipe and the concrete surface. Due to the convenient construction process, this bolted connector can be employed in prefabricated composite beams or bridges. Due to the different construction process, the mechanical behaviors of corrugated pipe grouting connectors may be different from those of traditional bolt connectors. At the same time, the current specifications for bolted connectors in composite structures are not suitable for bolt connectors. Therefore, investigations on the fundamental performance and design guidance of this advanced bolted shear connector are necessary.

In this paper, a systematic investigation was performed to understand the mechanism and performance of the proposed advanced connector. Referring to previous study conducted by Ding et al. [4] 22 static push-off tests were conducted considering the effect of the reserved hole constraint condition, the bolt dimension, and the diameter of the reserved hole. Corresponding simulation framework was further built and validated based on the test data. Influencing patterns of different parameters including concrete strength, bolt dimension, yield strength, bolt pretension, as well as length-to-diameter ratio of high strength bolts were discussed. On the basis of results, an innovative design approach for shear resistance calculation as well as load-slip relationship was proposed.

## 3. Experimental Research

### 3.1. Test Specimens

In order to investigate the performance of the proposed shear connectors with a high-strength bolt, which was widely used in the prefabricated steel-concrete beams, a group of push-off experiments on the high-strength bolt shear connectors were carried out according to the static standard push load. To study the behavior of strength, shear stiffness, and the failure modes, 22 specimens with three different design parameters were investigated. The specimens were designed and fabricated according to Eurocode 4 [19], and the variables included the reserved hole constraint condition (Figure 1b,e,f), the bolt diameters d, the slab hole diameters *D* and the hole diameter ration *D*/*d*. Thus, the specimens, that can be classified as cast-in-situ concrete slab composite push-off test specimens (Type 1) and precast concrete slab composite specimens (Types 4 and 5), were divided into three groups which were T1, T4, and T5. The first group denoted as group T1 had six specimens that were designed for cast-in-place slabs with no reserved hole. The T4 group had eight regular precast slabs with three bolt dimensions and two slab hole sizes. In addition, the last group, T5, was designed for precast slabs with embedded corrugated pipes which also included eight specimens. Detailed parameters and specimen conditions in each group are presented in Table 1. The *l*_E_ represents the depth at which high-strength bolts were embedded into the concrete slabs.

The T4 models had a regular precast slab with round spared holes. The slab holes were achieved by placing the steel tubes at desired locations before concrete casting. The steel tubes were removed after 2 h of concrete hardening. To ensure a good bonding between the precast concrete and the refilled material, the inner surfaces were carved with the same corrugated curves as in corrugated pipes. In the T5 group, the spared corrugated pipe holes were made by placing the corrugated pipes before concrete casting. Considering the compactness and rapid solidification of the in-fill material, non-shrinkage self-compacting cement based high strength pouring material (HPG) was chosen as the refill material in this study. The fabrication of the connector was undertaken at one side and then the other side. The high-strength bolts were bolted by applying pretension to the steel beam flanges in accordance with the GB 50017-2017 [22] at first, then concrete was casted on site (T1) or the precast concrete slab (T4/T5) after hoisting to the predetermined position of the steel beam for grouting. After the in-fill material hardened, the specimen was turned over and the same fabrication process was conducted again in the same way.

**Figure 2:**
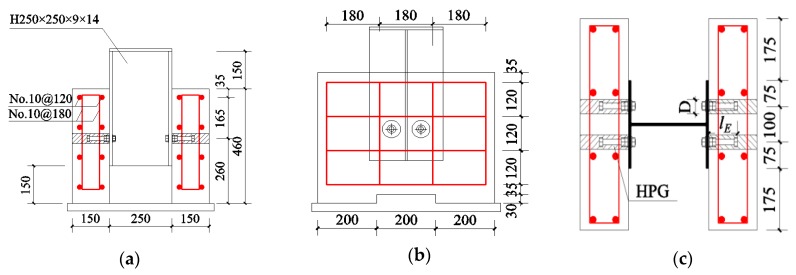
Details of the specimens (Units: mm): (**a**) front view; (**b**) side view; (**c**) top view.

Figure 2 gives detailed information of tested specimens. Those specimens consisted of a Chinese steel beam with a dimension of 250 mm × 250 mm × 14 mm × 9 mm (hw × bf × tf × tw). The beam flanges were connected by two concrete plates that were 460 mm wide, 600 mm long, and 150 mm thick, while high-strength bolts with diameters of 10, 12, or 16 mm were mounted on each flange.

### 3.2. Materials

The steel beam was made of Chinese Q235 steel with nominal strength of 235 MPa, and the 10 mm diameter hot-rolled ribbed bars HRB400 were used for the reinforcing bars. Tensile coupling tests were conducted for the steel beam, reinforcing bars, and high-strength bolt in accordance with the GB/T 228-2010 [23] before the test. Measured material properties are presented in Table 2. This study employed two types of concrete including C30 concrete (average 28-day target compressive strength justified as 33.7 MPa) which was used for the concrete slab, and the non-shrinkage self-compacting cement base high strength pouring material (HPG) which was used as the in-fill material for Type 4 and Type 5. The material properties of the concrete were measured by standard cube (150 mm × 150 mm × 150 mm) tests after 28 days of hardening according to the code GB/T 50081-2002 [24], as presented in Table 2.

### 3.3. Experimental Setup and Loading Procedure

**Figure 3:**
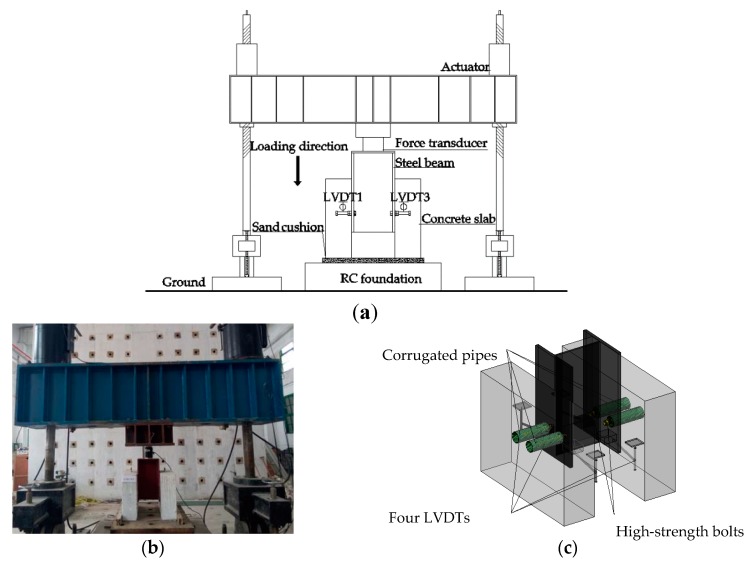
Test setup: (**a**) push-off test; (**b**) experimental setup photograph; (**c**) arrangement of linear variable displacement transducers (LVDTs).

The setup for the push-out test is shown in Figure 3. The push-out tests were implemented under a load capacity of 4500 kN and a stroke of ±200 mm in a hydraulic testing system. Specimens were fastened to a steel platform and then sat on a reinforced concrete (RC) base of the loading machine. Before the test, the set up and instrumentation were examined by a displacement controlled under cyclic loading conditions at a speed of 5 mm/min. Additionally, during the preloading process 15 cycles were performed with the amplitude ranging from 5% and 40% of expected failure load. During the real loading period, the vertical downward displacement load was employed at a constant rate of 0.3 mm/min until failure. Four linear variable displacement transducers (LVDTs) were applied in the measurement of the relative displacement between the steel beam and the concrete slab. The LVDTs were all mounted on the same horizontal position at the four corners of the specimen as illustrated in Figure 3a,c. No strain measurements were made in this study.

## 4. Results and Discussion

### 4.1. Load-Displacement Relationship

**Figure 4:**
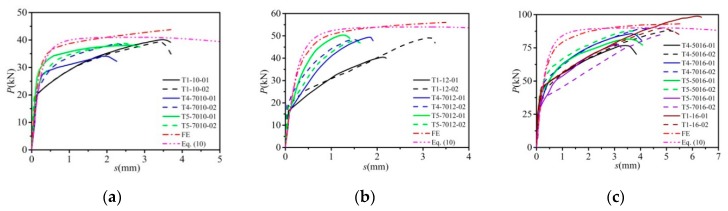
Load-displacement curves for push-off tests: (**a**) specimens of 10 mm; (**b**) specimens of 12 mm; (**c**) specimens of 16 mm.

The measured load-slip relation curves of the tested specimens is presented in Figure 4, and the longitudinal slip is presented as the averaged value for four LVDTs divided by the initial accumulated slip during the preloading process. The load-displacement curves generally included three parts, namely the linear elastic stage, plastic hardening stage, and the failure stage. At the initial period of loading, high shear resisting stiffness was displayed due to the mechanical friction between the slab and steel flange which resulted from the high pretension from the high-strength bolts. As the applied load increased, the friction diminished at the beam-slab interface, and the stiffness exhibited a decreasing tendency with the development of the slip. During the latter failure period, the slip increased rapidly till the specimens experienced failure. 

It can be clearly observed that the ultimate capacity of bolted shear connectors increases with the bolt diameter increase. The construction methods of concrete slab have little influence on the shear capacity of bolted shear connectors. For example, when the bolt diameter is the same as 12 mm, the mean ultimate capacity of T1, T4, and T5 specimens were 47.3, 48.9, and 49.1 kN, respectively. As the grouting material (HPG) and corrugated pipes on the bolt constraints are stronger, the shear stiffness of T5 specimens is higher than T4 and T1 specimens, but the maximum slip of the T5 specimens was smaller than T4 and T1 specimens. Detailed discussions are illustrated in the following section.

### 4.2. Failure Modes

**Figure 5:**
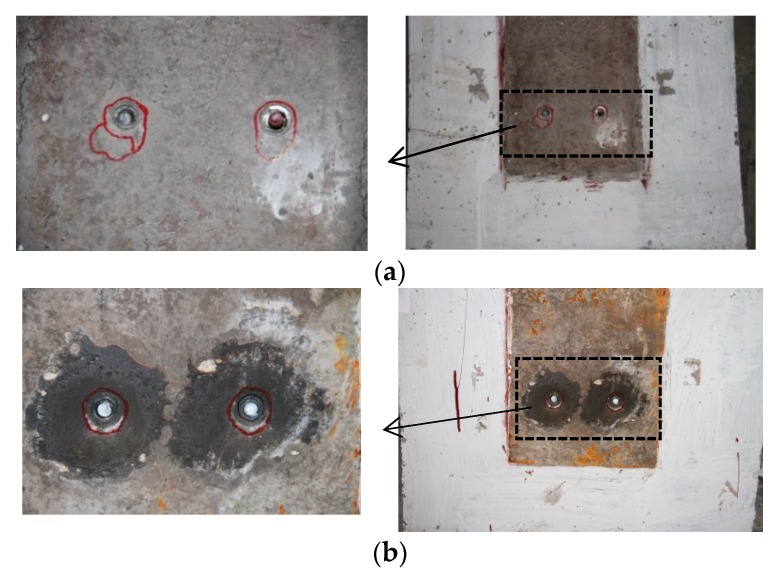

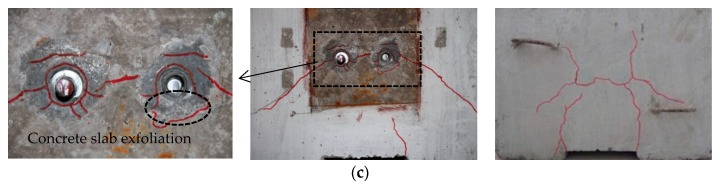
Failure modes: (**a**) 10 mm bolt and concrete slab (T1-10-01); (**b**) 12 mm bolt and concrete slab (T4-7012-02); (**c**) 16 mm bolt and concrete slab (T5-5016-01).

The failure of all test specimens was observed at the high strength bolts with factures developed at the junction of the steel beam and concrete slabs. Surface of the fractures all displayed obvious shear deformation. The failure modes of the three bolt dimension cases are shown in Figure 5 as well as the development of crack in the concrete slabs at the moment of bolt failure. At both the internal and external surfaces of the concrete slabs, no obvious cracks were observed in all specimens with 10 mm bolts (Figure 5a). Additionally, no obvious separation was presented between the concrete slabs and the beam flanges. For shear connectors with 12 mm bolts, there was separation between concrete slabs and beam flanges, but the obvious crack developments were rarely detected at the moment of separation for the majority of specimens (Figure 5b). Shear connectors with 16 mm bolts displayed concrete cracks in the failure mode. Cracks were observed at the bolt position extended downward towards both corners at the bottom side of the concrete slabs, exfoliation of a small amount of concrete was observed at the lower side of the bolt, and there was a clear separation between the concrete and the upper surface of bolts (Figure 5c). Compared to the T1 specimens, T4 and T5 specimens presented more crack developments in the prefabricated slabs around the reserved holes when the specimens failed. Moreover, due to the strong constraint of the corrugated pipe on the in filled concrete, specimens with corrugated pipes (T5) in comparison with those with normal reserved hole (T4) and cast-in-place specimens (T1) displayed less crack development and smaller bolt shear deformation.

### 4.3. Experimental Results Analysis

Summary of the shear stiffness, bearing capacity, and the maximum slip amount for each connector is shown in Table 3. *P*_u_ and *P*_u,m_ refer to the ultimate load and the mean value. *S*_u_ and *S*_u,m_ denote the maximum slip and the mean value. In this paper, the shear stiffness (*k*_0.4_) is the stiffness for each connector defined at the point with 40% ultimate load in the load-slip relation curves. Then in the following, the effect on shear capacity, shear stiffness, and the maximum slip produced by factors such as bolt diameter, the reserved hole constraint condition, and hole diameter ratio are discussed in detail.

#### 4.3.1. Influence of Bolt Diameter

**Figure 6:**
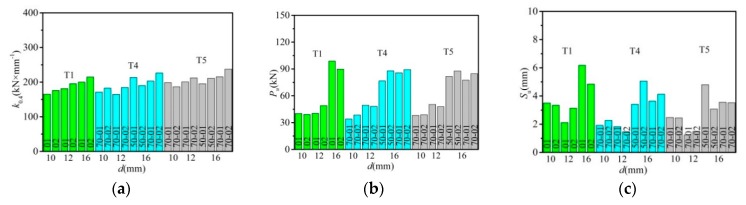
Effect of bolt diameter: (**a**) shear stiffness; (**b**) shear capacity; (**c**) the maximum slip.

Figure 6 shows the effect of bolt diameter on the shear stiffness, shear capacity, and the maximum slip given the same concrete slab. With the increase in bolt diameter from 10 to 16 mm, shear stiffness displayed an upward trend in the majority of specimens. As for shear capacity, with the increasing of bolt diameter, the ultimate load also increased. Under the equivalent reserved hole constraint condition, specimens with 16 mm bolts had higher average shear capacity than 10 mm bolt specimens. The strength ratios of the 16 mm bolt specimens were 137.5% for T1 specimens, 133.4% for T4 specimens, and 115.3% for T5 specimens, respectively. As bolt diameter increased, the maximum slip exhibited a decreasing tendency at first while had an upward trend during the later period.

#### 4.3.2. Influence of the Reserved Hole Constraint Condition

**Figure 7:**
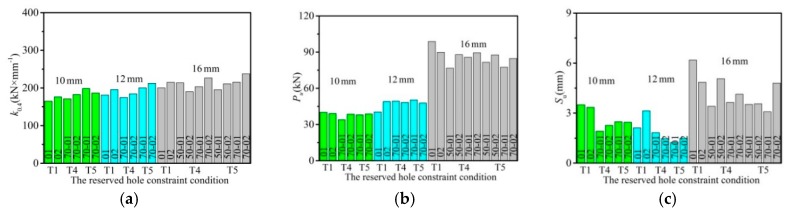
Effect of the reserved hole constraint condition: (**a**) shear stiffness; (**b**) shear capacity; (**c**) the maximum slip.

The results obtained are shown in Figure 7 in accordance with the reserved hole constraint condition. Under the condition that the bolt sizes of specimens were set as the same, the majority of T5 specimens primarily presented the maximum shear stiffness, except for individual specimens. The main failure mode as shown in specimens with bolt fractures, was that shear capacity was influenced little by the reserved hole constraint condition. While, the T4 and T5 specimens showed smaller maximum slip that resulted from the constraint from the corrugated pipe and HPG.

#### 4.3.3. Influence of Hole Diameter Ratio

**Figure 8:**
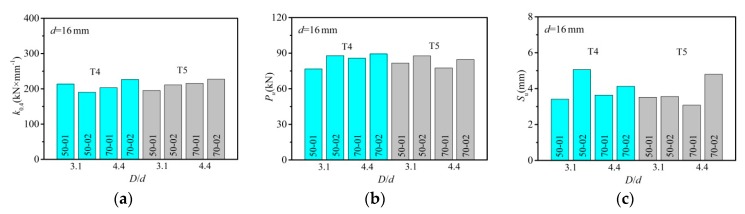
Effect of hole diameter ration (*D*/*d*): (**a**) shear stiffness; (**b**) shear capacity; (**c**) the maximum slip.

A comparison was made between specimens with the same diameter for detection of the shear stiffness, shear capacity, and the maximum slip under the same reserved hole constraint condition, however, at different hole diameter ratios (*D*/*d*). Given the results presented in Figure 8, which showed that with the increase of hole diameter ratio up to 4.4 from 3.1, the shear stiffness increased. To be more detailed, compared to the T4 specimen with *D*/*d* = 3.1, the average shear stiffness presented an increase by 6.5% in the T4 specimen with *D*/*d* = 4.4. In addition, with the increase of hole diameter ratio from 3.1 to 4.4, an increase of the average shear stiffness by 11.4% was observed in T5 specimens. In terms of shear capacity, the diameter ratio produced little influence. The maximum of T5 specimens slightly increased with the increase of diameter ratio.

## 5. Finite Element Model

### 5.1. General

As the shear capacity of high-strength bolt shear connectors was influenced little by the reserved hole constraint condition, the contact relationship among high-strength bolts, corrugated pipes, and grouting materials was complex. In this paper, a generic model without considering the corrugated pipes and the grouted materials was established and used to investigate the mechanical behaviors of the high-strength bolt shear connectors using the ABAQUS/Standard 6.12. The tests adopted most of components which were properly modeled to match the real condition, including concrete slab, high-strength bolt shear connectors, steel beam, and reinforced bars. During the test, the slip was found to be quite small between the nut and the thread, therefore, the nut and thread of the high strength bolt were merged and modeled as a single part in the numerical model.

**Figure 9:**
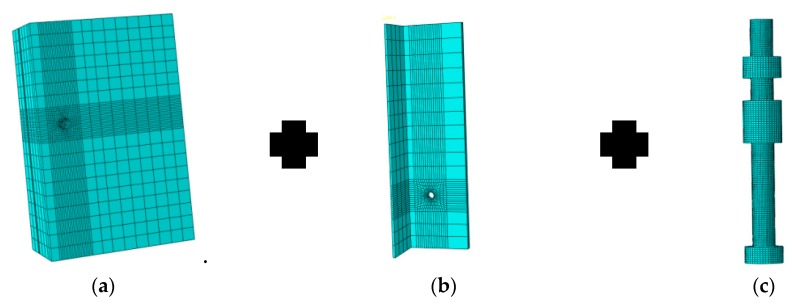

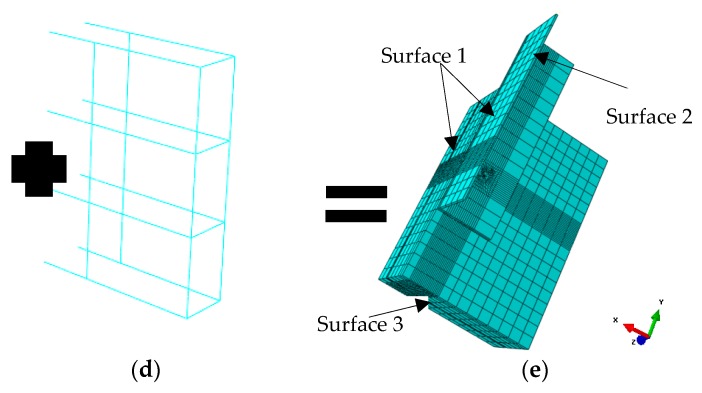
Finite element (FE) analysis model: (**a**) concrete; (**b**) steel beam; (**c**) high-strength bolt; (**d**) steel cage; (**e**) model.

An eight-node reduced integral solid element (C3D8R) with the hourglass control was used for modeling the concrete slab, steel beam, and high-strength bolts. Truss elements (T3D2) were applied to simulate the reinforced bars embedded in the concrete slab. Figure 9 shows the meshing details of the finite element (FE) model. As the construction is exactly symmetrical, only a quarter of the specimens and push-out arrangements were established. The round bars with equivalent section diameters were used to replace the thread and the bolt head. To ensure the accuracy of the stimulation with lower computational cost, the concrete slab was meshed with size of 25 mm, steel beam with 30 mm, the bolt connectors with 1.2 mm, regions around the concrete holes with 2.0 mm, and regions around the steel holes with 1.5 mm.

### 5.2. Material Models

The material constitutive models for concrete slab, steel beam and reinforcements that were used in this study referenced the work of Ding et al. [25]. The stress–strain relationship of concrete is described as follows:

(1) $$y=\left\{ \begin{array}{ll} \frac{Ax+(B-1)x^{2}}{1+(A-2)x+Bx^{2}} & x\leq 1\\ \frac{x}{\alpha_{1}{\left(x-1\right)}^{2}+x} & x>1 \end{array}\right.,$$

where *y* = *σ*/*f*_c_ and *x* = *ε*/*ε*_c_, σ, and ε are the stress and strain of the core concrete, *f*_c_ is the uniaxial compressive strength of concrete, *ε*_c_ is the strain corresponding with *f*_c_, *f*_c_ = 0.4*f*_cu_^7/6^, *ε*_c_ = 383*f*_cu_^7/18^ × 10^−6^, *f*_cu_ is the compressive cubic strength of concrete, with the parameters *A* = 9.1*f*_cu_^−4/9^ and *B* = 1.6(A − 1)^2^. The key material parameters dilation angle *ψ*, flow potential eccentricity e, biaxial/uniaxial compressive strength ratio *f*_b0_/*f*_c0_, and ratio K were adopted as 40°, 0.1, 1.225, and 2/3, respectively [26].

An elastic-plastic model was employed to interpret the constitutive behavior of the steel beam and reinforcements with the Von Mises yield criteria, Prandtl–Reuss flow rule, and isotropic strain hardening, and the stress–strain relationship is defined as follows:

(2) $$\sigma_{i}=\left\{ \begin{array}{l} E_{s}\varepsilon_{i}\\ f_{s}\\ f_{s}+0.46\%E_{s}(\varepsilon_{i}-\varepsilon_{st})\\ f_{u} \end{array}\right.\begin{array}{c} \varepsilon_{i}\leq \varepsilon_{y}\\ \begin{array}{l} \varepsilon_{y}<\varepsilon_{i}\leq \varepsilon_{\mathrm{st}}\\ \varepsilon_{\mathrm{st}}<\varepsilon_{i}\leq \varepsilon_{u} \end{array}\\ \varepsilon_{i}>\varepsilon_{u} \end{array},$$

where *σ*_i_ is the equivalent stress of steel, *ε*_i_ is the equivalent strain; *f*_s_ is the yield strength and *f*_u_ is the ultimate strength, *f*_u_ = 1.5*f*_s_; *E*_s_ is the elastic modulus; *ε*_y_ is the yield strain and *ε*_st_ is the hardening strain, *ε*_st_ = 12ε_y_; *ε*_u_ is the ultimate strain with the value of 120*ε*_y_.

A tri-linear model proposed by Loh et al. [27] was developed, in order to simulate the response of high-strength bolt material and the stress–strain curve is defined as follows:

(3) $$\sigma_{\mathrm{bt}}=\left\{ \begin{array}{l} E_{\mathrm{bs}}\varepsilon_{\mathrm{bt}}\\ 0.94f_{\mathrm{btu}}+0.86\%f_{\mathrm{btu}}/\varepsilon_{\mathrm{bty}}\times \\ f_{\mathrm{btu}} \end{array}\right.(\varepsilon_{\mathrm{bt}}-\varepsilon_{\mathrm{bty}})\begin{array}{c} \varepsilon_{\mathrm{bt}}\leq \varepsilon_{\mathrm{bty}}\\ \varepsilon_{\mathrm{bty}}<\varepsilon_{\mathrm{bt}}\leq 8\varepsilon_{\mathrm{bty}}\\ \varepsilon_{\mathrm{bt}}>8\varepsilon_{\mathrm{bty}} \end{array},$$

where *σ*_bt_ is the equivalent stress of high-strength bolt, *ε*_bt_ is the equivalent strain; *f*_btu_ is the ultimate strength; *ε*_bty_ is the yield strain; *E*_bs_ is the elastic modulus.

### 5.3. Boundary Conditions

The symmetric boundary was employed to the symmetry planes of the specimens. As shown in Figure 9, nodes at the symmetry plane of the steel beam flange and the concrete slab (Surface 1) were inhibited with X direction translation and Y and Z axes rotation (UX = URY = URZ = 0). All nodes at the middle plane of the steel beam web (Surface 2) were restrained at the Z direction translation and X and Y axes rotation (UZ = URX = URY = 0). Moreover, nodes at the bottom plane of the concrete slab (Surface 3) were controlled at the Y direction translation to resist the compression load.

### 5.4. Interaction and Constraint Conditions

Test results presented the shear resisting process involved the separation between high strength bolts and assembled slab or beam flanges, and the frictional slipping behaviors at the interfaces between the beam flange and concrete slab. Then the contact bonding and interactions must be considered during the simulation. The surface-to-surface contact interaction in ABAQUS was applied at the above-mentioned contact interfaces. The HARD contact was used normally in the direction toward the interface plane, and the PENALTY option was adopted for the tangential response. The friction coefficients for the contact between the steel and concrete components was determined as 0.45 and 0.25 for all of the other interactions [16,17]. Embedded constrains were also implemented, in order to model the constraint between the concrete slab and the bars. In terms of concrete slabs, no relative slip or debonding of the reinforcement, were taken into account.

### 5.5. Load Application and Analysis Steps

The analysis mainly consisted of two steps. In the first step, the BOLT LOAD function available in ABAQUS was used for implementing the pretension forces to the bolt connectors. In the second step, following the test, push-down load was applied on the top surface of the steel beam.

### 5.6. Comparison and Discussion of Results

Dashed lines, as shown in Figure 4, are the load-slip curves obtained from the FE simulations. Due to the simplification of the model, the load-slip curves of the FE results have no significant descend stage. In this study, *P*_u_ was obtained considering that failure was reached when the concrete reached its ultimate stress, or the high-strength bolts reached their yield stress. As there was no bolt thread in the FE models, then the bolt models were in fact closer to being traditional studs. Therefore, the FE curves for bolt connectors displayed similarity to headed stud shear connectors in the load-slip characteristics, especially in plastic hardening stage. During the elastic-plastic stage, shear stiffness of the specimens presented significant degeneration resulting from screw thread, which can be further attributed to the non-compactness of the surrounding concrete during casting, especially for T1 specimens. As a whole, the upper limit of shear strengths can be predicted using the FE simulations in push-out tests. Additionally, T5 specimens displayed the maximum matching degree between the FE curves and test data.

**Figure 10:**
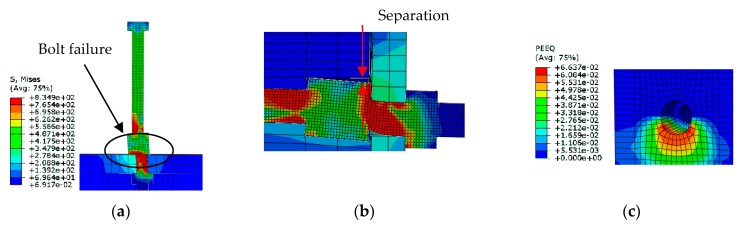
Finite element failure mode of 16 mm high-strength bolt shear connector (Units: MPa): (**a**) bolt failure; (**b**) separation between the concrete and bolt; (**c**) concrete exfoliation.

The Von Mises stress and equivalent plastic strain conditions at the failure load in specimen with 16 mm high-strength bolt shear connector are given in Figure 10. The maximum stress was observed at the interface between the concrete and the steel beam in high strength bolt with a value about 835 MPa, reaching the ultimate strength of a 16 mm bolt. This high stress indicated the bolt failure in the shear connector and this failure mode agreed with the experimental results (Figure 5). Moreover, the concrete elements got peeled off at regions around the bottom side of high strength bolt (Figure 10c), which also was identical to the experimental observations given in Figure 5e.

### 5.7. Parametric Study

In order to further investigate the proposed high-strength bolt shear connectors (T5), parametric studies were conducted using the simplified FE model, through the push-out tests developed above. The patterns of influences on the ultimate shear bearing capacity produced by concrete strength and the bolt dimension, yield strength, length-to-diameter ratio, and the pretension of high strength bolts were investigated and discussed.

#### 5.7.1. Effect of Bolt Diameter and Concrete Strength

**Figure 11:**
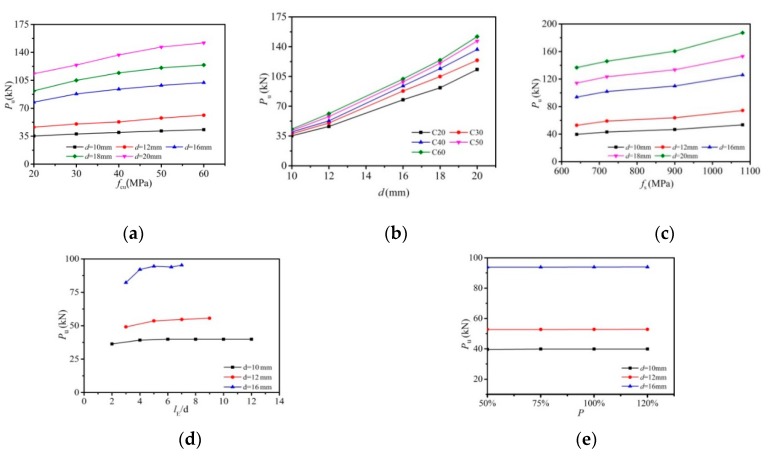
Main factors for shear capacity of high-strength bolt connectors: (**a**) effect of concrete strength; (**b**) effect of bolt diameter; (**c**) effect of bolt yield strength; (**d**) effect of length-to-diameter ratio; (**e**) effect of bolt pretension.

In this section, the FE model was employed with 640 MPa bolt yield strength and embedded length (*l*_E_) of 100 mm. The model was used to investigate the influence of bolt diameter and the nominal concrete strength *f*_cu_ on shear resistance with the former varied from size of 10 to 20 mm and the latter ranging from 20 to 60 MPa. The calculated curves are presented in Figure 11a,b. As presented in the results, the ultimate shear resistance increased with the increase of either the concrete strength or bolt diameter. With the increase of concrete strength from 20 to 60 MPa, increases were exhibited in the ultimate shear capacity by 22% for the 10 mm bolt connector, 32% for the 12 mm bolt connector, 31% for the 16 mm bolt connector, 35% for the 18 mm bolt connector, and 34% for the 20 mm bolt connector. Figure 11b shows that at the concrete strength of 40 MPa, the ultimate shear capacity was increased by 136% with the bolt diameter raised from 10 to 16 mm, and was further increased by 46% as the bolt diameter was lifted from 16 to 20 mm.

#### 5.7.2. Effect of Bolt Yield Strength 

The influence of bolt yield strength was investigated by raising the nominal yield strength from 640 to 1080 MPa, the nominal concrete strength *f*_cu_ set as 40 MPa, and the embedded length settled as 100 mm. As presented in Figure 11c, the comparison indicated that the ultimate shear resistance almost linearly increased with the increase of the bolt yield strength. As the bolt yield strength was raised from 640 to 1080 MPa, the ultimate shear capacity presented an increase by 34.5% for the 10 mm bolt connector, 45.7% for the 12 mm bolt connector, 34.2% for the 16 mm bolt connector, 34.0% for the 18 mm bolt connector, and 36.9% for the 20 mm bolt connector.

#### 5.7.3. Effect of Length-to-Diameter Ratio of the Bolt

Three different diameters, 10, 12, and 16 mm, were chosen to verify the effect of length-to-diameter ratio of the bolt. With the concrete strength and bolt yield strength set as 40 and 640 MPa, length to diameter ratios varied from 2 to 12. As shown in Figure 11d, the numerical results demonstrated that below the ratio of 4, the ultimate shear capacity increased as the ratios increased. While at a ratio of greater than 4, further increase was not presented in the shear resistance. This influencing pattern is similar to that in the work of Pavlović et al. [20].

#### 5.7.4. Effect of Bolt Pretension

Figure 11e exhibits the influence of bolt pretension on the ultimate shear capacity. The abscissa P presents the pretension recommended in the design code GB 50017-2017 [22] for steel structures. It was found that the bolt pretension has little impact on the ultimate shear capacity of high strength bolt connectors, which is similar to conclusions drawn by Zhang et al. [28].

#### 5.7.5. Summary

The findings can be summarized as follows, the ultimate shear capacity of high-strength bolt connectors was improved with the increase of bolt diameter, yield strength of bolt, and concrete strength. For the proposed high strength bolt shear connector, the ultimate shear resistance was most significantly increased by increase in the bolt diameter.

## 6. Ultimate Shear Bearing Capacity Calculation

### 6.1. Establish Formula

A total of 72 FE models were analyzed in the parametric FE studies. Based on both experimental and numerical results in the parametric studies, the influencing trend was taken to carry out a non-linear regression and to work out the expression of the shear capacity of proposed shear connector. The equation can be written as:

(4) $$P_{u}=0.23d^{1.78}{f_{cu}}^{0.29}(0.0007f_{s}+0.53),$$

where *f*_cu_ is the mean value of cubic actual compressive strength of concrete; *f*_s_ is the bolt yield strength and *d* is the diameter of bolt shank. The units for the *P*_u_, *d*, *f*_cu_, *f*_s_ in the expression are kN, mm, MPa, MPa.

### 6.2. Comparison with Different Standard Provisions

At present, there are rare reports about relevant formulas for high-strength bolt shear connectors used in steel-concrete composite beams in various countries. Therefore, the shear capacities calculated with the proposed formula were compared to the strengths obtained from tests and numerical analyses. Additionally, accuracy comparisons were performed between the proposed formula and design equations for headed stud connectors in GB 50017-2017 [22], AISC [29], EC4 [19], together with the calculation method proposed by Kwon et al. [14] and Liu et al. [16].

In the design code GB 50017-2017 [22] for steel structures, the stud shear resistance was determined by

(5) $$P_{u}=0.43A_{sc}\sqrt{E_{c}f_{c}}\leq 0.7A_{sc}\gamma f_{u},$$

where *P*_u_ = ultimate strength of welded stud shear connectors, *γ* is the ratio of the minimum tensile strength to yield strength of the stud. Since the bolt fracture was extended to the surface of the thread part in the tests, we have *A*_sc_ = 0.781A_s_ here in the paper. *A*_s_ is the gross cross-sectional area of the bolt shear connector.

In AISC [29], the nominal shear resistance of headed stud shear connector that embedded in a concrete deck was governed by

(6) $$P_{u}=0.5A_{sc}\sqrt{E_{c}f_{c}^{\prime }}\leq A_{sc}f_{u},$$

where *f*_c_^’^ is the cylinder compressive strength of concrete and was calculated according to Ding et al. [25].

While, EC4 [19] adopted a similar approach to determine the ultimate resistance of headed stud connectors, in which the minimum value obtained from the following two equations was taken as the ultimate strength.

(7.1) $$P_{u}=0.29\alpha d^{2}{\left(E_{c}f_{c}^{\prime }\right)}^{0.5}/\gamma_{v},$$

and

(7.2) $$P_{u}=0.8A_{sc}f_{u}/\gamma_{v},$$

where *α* is aspect ratio factor, when 3 ≤ *h*_sc_/*d* ≤ 4, α = 0.2(*h*_sc_/*d* + 1); when *h*_sc_/*d* ≤ 4, *α* = 1; *γ*_v_ is a partial factor which is equal to 1.25.

Kwon et al. [14] suggested an equation (Equation (8)) to calculate the ultimate strength of post-installed shear connectors *V*_u_ under static loading

(8) $$P_{u}=0.5A_{sc}f_{u}.$$

Then, Liu et al. [16] modified the coefficients used in Equation (8) to achieve a new equation (Equation (9)) to predict the ultimate strength of the high-strength friction-grip bolt (HSFGB) shear connector *V*_u_.

(9) $$P_{u}=0.66A_{sc}f_{u}.$$

The units for the variables in the above expressions are N and mm.

**Figure 12:**
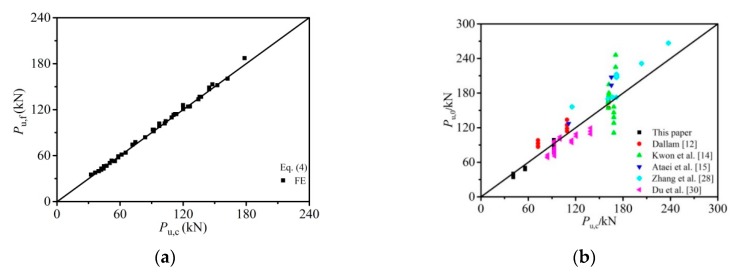
Comparison of the predicted bearing capacities with the FE example results and the test results: (**a**) comparison of the predicted bearing capacities with the FE example results; (**b**) comparison of the predicted bearing capacities with the test results.

The comparison between shear strength data and the results predicted from different equations (Equations (4)–(9)) was made, as shown in Figure 12a and Table 4. Apart from the test data and FE simulations given in this paper, the shear capacities of 86 push-out tests from other existing studies [12,14,15,28,30] were also involved in the comparison. *P*_u,f_, *P*_u,0_, and *P*_u,c_ represent the FE data, experimental data, and the calculated shear capacities, respectively. It was seen in results that the Equation (4) proposed in this paper produced an accurate prediction on the shear strength in high strength bolt connectors (with the mean error *µ* = 1.010 and a variation coefficient *η* of 0.025 for 45 FE data).

The comparison between predicted capacities and the test results is presented in Figure 12b and Table 5. The predicted values from Equation (4) were in a good agreement with the test results (*µ* = 0.988, *η* = 0.161 for 86 push-off tests). The calculation method in GB 50017-2017 [22], AISC [29], EC4 [19], and formulas proposed by Kwon et al. [14] are relatively conservative (*µ* = 1.470, *η* = 0.501 for code GB 50017-2017 [22], *µ* = 1.215, *η* = 0.224 for code AISC [29], *µ* = 1.376, *η* = 0.275 for code EC4 [19] and *µ* = 1.374, *η* = 0.274 for Equation (8)). The formula (Equation (9)) suggested by Liu et al. [16] is also available for the prediction of the published experimental data (*µ* = 1.041, *η* = 0.207), but with less accuracy than the formula proposed in this study.

## 7. Load-Slip Relationship Calculation

### Formula Development

Referring to previous study conducted by Ding et al. [4], the full load-slip curve was proposed for high-strength bolt shear connectors in push-out tests and the curve can be expressed as:

(10) $$y^{\prime }=\left\{ \begin{array}{l} \frac{A_{1}x^{\prime }+(B_{1}-1){x^{\prime }}^{2}}{1+(A_{1}-2)x^{\prime }+B_{1}{x^{\prime }}^{2}}\\ \frac{x^{\prime }}{0.15{\left(x^{\prime }-1\right)}^{2}+x^{\prime }} \end{array}\right.\begin{array}{c} x^{\prime }<1\\ x^{\prime }>1 \end{array},$$

where *y*’ = *P*/*P*_u_, *x*’ = *s*/*s*_u_, *B*_1_ = 1.6(*A*_1_ − 1)^2^. 

The bond stiffness *k*_s_ is defined as the secant stiffness at the point with 40% ultimate load in the load-slip curve, and based on the experimental and numerical results, the *k*_s_ can be written as:

(11) $$k_{s}=(0.23d+\frac{91.2}{d}-7.15)P_{u}.$$

The unit is kN/mm.

**Figure 13:**
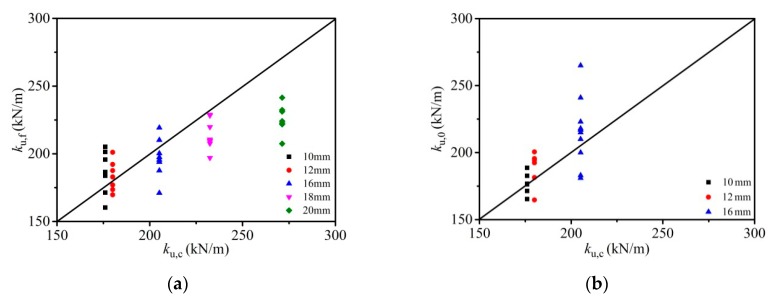
Comparison of predicted bond stiffness with the FE example results and the test results: (**a**) comparison of predicted bond stiffness with the FE example results; (**b**) comparison of predicted bond stiffness with the test results.

Figure 13 shows the comparison of predicted bond stiffness with the test results and the FE example results. The *k*_u,0_, *k*_u,f_, and *k*_u,c_ denote the experimental data, the FE data, and the calculated bond stiffness, respectively. Equation (11) suggested in this paper proved to exhibit a good agreement on predicted bond stiffness, especially in the small diameter (10~16 mm) bolt shear connectors (with the mean error *µ* = 0.965 and a variation coefficient *η* =0.071 for 22 test results, and the *µ* = 1.049 and *η* = 0.094 for 45 FE results).

There is much correlation between the slip displacement *s*_u_ corresponding to the peak load and the diameter of the high-strength bolt, and the formula can be written as:

(12) $$s_{u}=0.3d+0.21.$$

The unit is mm.

The ascending parameter *A*_1_ is defined as the ratio of bond stiffness to peak secant stiffness and can be expressed as:

(13) $$A_{1}=(0.3d+0.21)(0.23d+\frac{91.2}{d}-7.15).$$

**Figure 14:**
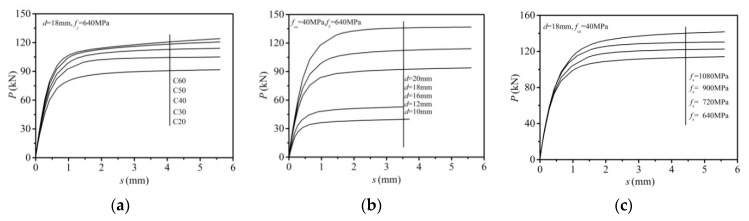
Load-slip curves of bolt shear connector: (**a**) concrete strength; (**b**) bolt diameter; (**c**) yield strength of bolt.

Figure 14 exhibits the load-slip curves of the bolt shear connector with different concrete strength, bolt diameter, and bolt yield strength on the basis of the FE calculation results of the parameter analysis of Section 5.7.

As illustrated in Figure 4, based on comparison of predicted results from the finite element results and Equation (10), and the load-slip curves of the push-out tests. It can be concluded that the proposed equation displayed a good agreement with the test and FE results. Consequently, the expression proposed in this paper can be recommended for the prediction on the load-slip relationship of per high-strength bolt shear connector, especially for the type of T5.

## 8. Conclusions

In this paper, a new high strength bolt shear connector was proposed with the corrugated pipe hole serving as the grout hole in the prefabricated slab. Experimental studies and FE simulations were conducted to investigate the shear resisting mechanism and strengths of the proposed connector. The objective of this work is to better understand the influences of main characteristics such as bolt diameter, yield strength, length-to-diameter ratio, and pretension as well as the concrete strength on the ultimate shear capacity, therefore, extensive parametric studies were also performed. The tests and numerical analysis made in this work led to the following conclusions.

1. The primary failure mode of the push-out tests was bolt failure. Additionally, the process of failure and crack developing was affected by the bolt diameter and the reserved hole constraint condition. The inhibition of crack developments in a concrete slab can be achieved by using precast concrete slab with corrugated pipes.
2. Experimental results demonstrated that bolt connectors with corrugated pipe had higher shear stiffness than normal reserved hole types and cast-in-place slab specimens. The ultimate shear capacity of the bolt connector was mainly influenced by the bolt diameter.
3. The developed numerical model produced satisfactory predictions of the behavior and ultimate shear capacity for the push-out tests on the bolted shear connector. The high strength bolt pretension presented little correlation to the shear capacity on the basis of FE simulations.
4. Based on the experimental data and numerical results, a new formula was proposed for calculation of the shear capacity of bolted shear connectors. The accuracy was discussed through the comparison to design code in GB 50017-2017, AISC, EC4, and the experimental and numerical data in this study and extracted from relative references. The proposed calculation method exhibited a better prediction to the experimental results than other formulas. By modifying the parameters, the load-slip curve of welded stud shear connectors proposed by Ding, can describe the load-slip relationship of bolted shear connectors, especially for T5 specimens, reasonably well.

## Figures and Tables

**Table 1:** Key information of specimens.

Specimen	*d* (mm)	*l*_E_ (mm)	*D* (mm)	*D*/*d*	Reserved Hole Constraint Condition
T1-10-01,02	10	100	-	-	Type 1
T1-12-01,02	12	100	-	-	Type 1
T1-16-01,02	16	100	-	-	Type 1
T4-5016-01,02	16	100	50	3.1	Type 4
T4-7016-01,02	16	100	70	4.4	Type 4
T4-7012-01,02	12	100	70	5.8	Type 4
T4-7010-01,02	10	100	70	7.0	Type 4
T5-5016-01,02	16	100	50	3.1	Type 5
T5-7016-01,02	16	100	70	4.4	Type 5
T5-7012-01,02	12	100	70	5.8	Type 5
T5-7010-01,02	10	100	70	7.0	Type 5

**Table 2:** Material properties of concrete, steel, and bolt.

Material	*f*_cu_ or *f*_y_ (MPa)	*f*_u_ (MPa)	*E*_s_ or *E*_c_ (MPa)
Concrete	33.7	NA	3.1 × 10^4^
HPG	67.0	NA	3.7 × 10^4^
Steel	256.4	457.1	2.03 × 10^5^
No. 10 bars	456	606	1.76 × 10^5^
10 mm bolt	696	877	2.08 × 10^5^
12 mm bolt	676	852	2.08 × 10^5^
16 mm bolt	663	835	2.08 × 10^5^

**Table 3:** Test results for each per connector specimen.

Specimens	Concrete Slab Type	*D*/*d*	*P*_u_ (kN)	*P*_u,m_ (kN)	*S*_u_ (mm)	*S*_u,m_ (mm)	*k*_0.4_ (kN × mm^−1^)	*k*_0.4,m_ (kN × mm^−1^)
T1-16-01	In situ	-	98.8	94.3	6.18	5.52	200.21	207.61
T1-16-02		-	89.8	-	4.85	-	215.00	-
T1-12-01		-	45.4	47.3	2.12	2.63	181.37	188.44
T1-12-02		-	49.1	-	3.14	-	195.50	-
T1-10-01		-	45.1	42.2	3.50	3.43	165.30	170.78
T1-10-02		-	39.2	-	3.35	-	176.62	-
T4-5016-01	Precast	3.1	76.7	82.3	3.41	4.24	213.63	201.92
T4-5016-02		3.1	87.9	-	5.06	-	190.20	-
T4-7016-01		4.4	85.7	87.6	3.64	3.89	203.34	214.97
T4-7016-02		4.4	89.4	-	4.13	-	226.60	-
T4-7012-01		5.8	49.4	48.9	1.83	1.65	174.67	179.59
T4-7012-02		5.8	48.3	-	1.47	-	184.50	-
T4-7010-01		7.0	34.1	36.4	1.92	2.10	171.35	177.04
T4-7010-02		7.0	38.6	-	2.27	-	182.72	-
T5-5016-01	Precast with corrugated pipe	3.1	81.6	84.7	3.52	3.54	195.38	203.17
T5-5016-02		3.1	87.7	-	3.56	-	210.96	-
T5-7016-01		4.4	77.5	81.1	3.08	3.94	215.51	226.39
T5-7016-02		4.4	84.7	-	4.80	-	237.26	-
T5-7012-01		5.8	50.3	49.1	1.24	1.37	200.54	206.50
T5-7012-02		5.8	47.8	-	1.49	-	212.45	-
T5-7010-01		7.0	38.1	38.5	2.48	2.47	198.67	192.70
T5-7010-02		7.0	38.8	-	2.45	-	186.72	-

**Table 4:** Comparison between the calculated results of the 45 finite element examples and equations.

	*P*_u,f_/*P*_u,(4)_	*P*_u,f_/*P*_u,(5)_	*P*_u,f_/*P*_u,(6)_	*P*_u,f_/*P*_u,(7)_	*P*_u,f_/*P*_u,(8)_	*P*_u,f_/*P*_u,(9)_
Mean (*µ*)	1.010	1.387	1.316	1.778	1.595	1.208
Coefficient of variation (*η*)	0.025	0.157	0.174	0.177	0.439	0.439

**Table 5:** Comparison between predicted bearing capacities and test results.

Reference	Number	*P*_u,0_/*P*_u,f_		*P*_u,0_/*P*_u,(4)_		*P*_u,0_/*P*_u,(5)_		*P*_u,0_/*P*_u,(6)_		*P*_u,0_/*P*_u,(7)_		*P*_u,0_/*P*_u,(8)_		*P*_u,0_/*P*_u,(9)_	
		*µ*	*η*	*µ*	*η*	*µ*	*η*	*µ*	*η*	*µ*	*η*	*µ*	*η*	*µ*	*η*
This paper	22	0.908	0.077	0.908	0.076	1.231	0.097	1.191	0.097	1.611	0.097	1.378	0.081	1.044	0.081
[12]	12	1.017	0.059	1.133	0.104	1.386	0.177	1.324	0.186	1.400	0.186	1.718	0.109	1.302	0.109
[14]	14	-	-	1.004	0.207	1.434	0.200	1.379	0.200	1.459	0.199	1.085	0.208	0.822	0.208
[15]	3	1.115	0.027	1.158	0.012	1.377	0.013	1.079	0.013	1.205	0.013	1.715	0.111	1.299	0.111
[28]	11	-	-	1.163	0.084	2.624	0.134	1.389	0.091	1.470	0.090	1.614	0.109	1.223	0.109
[30]	24	-	-	0.886	0.094	1.239	0.091	1.209	0.092	1.083	0.096	1.237	0.127	0.937	0.127
All	86	-	-	0.988	0.161	1.470	0.501	1.215	0.224	1.376	0.275	1.374	0.274	1.041	0.207

## Nomenclature

*l*_E_	depth of bolt shank inside concrete	*P*_u_	ultimate shear strength
*d*	high-strength bolt diameter	*P*_u,m_	the mean value of ultimate load
*D*	precast concrete slab hole	*S*_u_	maximum slip
*f*_cu_	compressive cube strength of concrete	*S*_u,m_	the mean value of maximum slip
*f*_c_	compressive strength of concrete	*A*_s_	gross cross-sectional area of bolt
*f*_c_^’^	compressive cylinder strength of concrete	*A*_sc_	effective cross-sectional area of bolt
*f*_y_	yield strength of steel	*α*	aspect ratio factor in Eurocode 4
*f*_s_	yield strength of bolt	*γ*	yield ratio of the tensile strength to the yield strength of bolt
*f*_u_	ultimate strength of steel	γ_v_	partial factor in Eurocode 4
*f*_btu_	ultimate strength of high-strength bolt	*P*_u,f_	ultimate shear strength of finite element result
*E*_s_	elastic modules of steel	*P*_u,0_	ultimate shear strength of experiment result
*E*_bs_	elastic modules of high-strength bolt	*P*_u,c_	ultimate shear strength of calculate result
*E*_c_	elastic modules of concrete	*P*_u,(__4)_	ultimate shear strength of the bolt according to Equation (4)
